# Evaluation of the implementation of the EWARS Mobile epidemiological surveillance tool in Sudanese refugee camps in Eastern Chad: a retrospective and population-based surveillance study

**DOI:** 10.3389/fepid.2025.1604446

**Published:** 2025-07-23

**Authors:** Stephane Tewo, Balde Thierno, Freddy M. Banza, Idriss M. Mahamat, N'dri K. Eric-Didier, Djinguebey N. Raoul, John Otokoye Otshudiema, Castilla Echenique Jorge, Moussa Brahimi, Djoumbarina Maina, Evers Egmond, Marcel Woung, Kazuki Shimizu, Boris I. Pavlin, Jacques L. Tamuzi, Patrick D. M. C. Katoto, Charles S. Wiysonge, Blanche-Philomene Melanga Anya

**Affiliations:** ^1^ WHO Country Office, N’djamena, Chad; ^2^ Emergency Preparedness and Response, World Health Organization (WHO), African Regional Office (AFRO), Dakar, Senegal; ^3^ Department of Health Emergencies Intervention, World Health Organization, Headquarters, Geneva, Switzerland; ^4^ Incident Management Team, Ministry of Health, N’djamena, Chad; ^5^ Department of Alert and Response Coordination, World Health Organization, Headquarters, Geneva, Switzerland; ^6^ Department of Global Health, Faculty of Medicine and Health Sciences, Division of Epidemiology and Biostatistics, Stellenbosch University, Cape Town, South Africa; ^7^ Office of the President and CEO, South African Medical Research Council, Cape Town, South Africa; ^8^ Department of Medicine, Centre for Tropical Diseases and Global Health, Catholic University of Bukavu, Bukavu, Democratic Republic of the Congo; ^9^ Cochrane South Africa, South African Medical Research Council, Cape Town, South Africa; ^10^ World Health Organization Regional Office for Africa, Brazzaville, Democratic Republic of Congo

**Keywords:** EWARS, surveillance, vulnerable children, refugees, outbreaks, humanitarian crisis, Sudan, Eastern Chad

## Abstract

**Background:**

The escalation of the conflict in Sudan has created a major humanitarian challenge for neighboring countries, especially in the Eastern regions of Chad. This humanitarian setting's health needs are unique in that they are more vulnerable to both outbreak-prone disease and a lack of essential services. To address these challenges, the World Health Organization has supported implementing the Early Warning Alert and Response System (EWARS) Mobile. The purpose of this study was to evaluate the application of the EWARS Mobile epidemiological surveillance tool in Sudanese children’s refugees in Eastern Chad.

**Methods:**

This was a retrospective and population-based surveillance study that provided an overview of the pattern of cases and deaths in time and space related to potential outbreaks.

**Results:**

In total, 1,645 alerts were reported among children in vulnerable provinces of Quaddai, Sila, and Wadi Fira. There were 41,738 alerted cases and 236 deaths, for a 0.56% projected fatality rate. The EWARS Mobile successfully reported alerted increases in cases of acute flaccid paralysis (AFP), acute jaundice syndrome (AJS), acute respiratory infection (ARI), acute watery diarrhea in children (AWD), measles, meningitis, diphtheria, neonatal tetanus (NT), dengue, dysentery, and atypical events in vulnerable children in time and space. Case reporting, alert recording, and weekly reporting were completed successfully at all levels (camps, district, zone, and province) (≥80% completion rate). In contrast, the timeliness of alert reporting, weekly reporting, and probable outbreaks did not perform well across levels (≥80% timeliness rate). Epidemic curves indicated multiple probable outbreak types, characterized by a point source (AJS and AWD under 5 years), common source (AWD in 5 years and above), propagated source (ARI and dysentery), and intermittent source (AFP, measles, meningitis, diphtheria, NT, and unusual events). The sensitivity and positive predictive value were estimated at 81% (79%–83%) and 72.0% (68%–75%), respectively.

**Conclusions:**

The EWARS Mobile is a practical solution for Eastern Chad provinces to implement throughout the pre-epidemic and outbreak periods in vulnerable children in this severe humanitarian crisis. However, efforts should be made to improve timeliness indicators at all subnational levels and incorporate alarm indicators.

## 1 Background

This year, at least 63 million, or 45% of the 141 million people across Africa, including refugees, are in need of humanitarian aid (1). By November 2024, over 20 million people in the region had been internally displaced in Sudan, the world's worst displacement catastrophe, accounting for 57% of the total (1). Furthermore, more than 5.6 million individuals are hosted as refugees or asylum seekers within the region (1). The Sudan crisis has created a major humanitarian challenge for neighboring countries, especially in the eastern regions of Chad. According to the Chadian government, 910,000 refugees and returnees could have arrived in Chad by the end of 2024 (2). In this humanitarian setting, health needs are unique in a way that they are more vulnerable to both outbreak-prone disease and lack of essential services. The vulnerability of such people in such a setting will span from water, sanitation, and hygiene (WASH)-related diseases including diarrheal disease, respiratory disease, and vaccine preventable disease (3). They are prone to the new hosting environmental health issues as well. Such vulnerability will call up on a comprehensive service of outbreak prevention and control, and continuation of essential health services (3). This humanitarian situation is exacerbated by the epidemics of dengue fever, measles, chickenpox, and hepatitis E (4). This humanitarian crisis may generate numerous risk factors that facilitate the emergence and transmission of communicable disease outbreaks, including food insecurity, the gradual erosion of livelihoods, and the disruption or collapse of preventive and curative health services, as well as access to safe water and sanitation. In addition, mass displacement of individuals into organized or camp-like settlements or neighboring host communities heightens the risk of overcrowding, abrupt loss of livelihoods, and/or swift environmental changes resulting from natural disasters, possibly also triggering infectious disease outbreaks (5–7). During the rainy season in Ouadi province, the health situation may be distressing because of the torrential rains, which are making it difficult to move temporary watercourses and provide adequate healthcare to refugees (4). A survey conducted in Eastern Chad revealed that access to health services is estimated at 39% and available assistance for refugees is estimated at 18% (5). These refugees live in numerous formal and informal camps in health districts in the provinces of Ennedi East, Ouaddaï, Sila, and Wadi Fira. In the camps, access to essential health services is disrupted due to difficult physical access and limited human and material resources. Malaria, acute respiratory infections (ARI), malnutrition, and acute watery diarrhea remain the most common pathologies.

The actual humanitarian crisis in Eastern Chad needs a multidisease surveillance system; a limited degree of integration and coordination may be required for efficiency (8). A systematic strategy is required to enhance national surveillance systems in Eastern Chad by prioritizing diseases for surveillance, conducting thorough assessments of current systems, formulating action plans for system improvement, executing these plans, and performing monitoring and evaluation (8). The urgent need for effective disease surveillance and rapid response mechanisms in such emergencies has become increasingly evident. To address these challenges, the World Health Organization (WHO) has launched the deployment of Early Warning Alert and Response System (EWARS) Mobile, an advanced early warning and response system tailored to emergencies. EWARS Mobile is a simple and cost-effective way to set up a disease early warning and response system. It is designed to improve disease outbreak detection and response in emergency settings, such as in countries in conflict or following a natural disaster (9). The deployment and implementation of the EWARS Mobile for the Sudan refugee crisis involve a multistep process, building on past experiences and adapting to current needs. Furthermore, the system is not limited to disease monitoring; it also encompasses monitoring service delivery, thus providing a holistic approach to health protection in crisis scenarios. At the same time, national surveillance systems may be underperforming, disrupted, or nonexistent during emergencies, which may adversely impact and delay the detection of and response to outbreaks (5, 10, 11). Studies have shown that the EWARS Mobile is a pragmatic solution for countries to mount during the preoutbreak phase as an effective outbreak response defined by alarm signals (12). Furthermore, this tool performed well (13) in terms of effectiveness and cost-effectiveness (14) and timely disease outbreak prediction (15). However, the prediction validity varied substantially across different types of diseases and settings and appeared less optimal in low endemic settings (15). EWARS Mobile has since been adapted and expanded to effectively respond to emerging health crises. However, scant research has addressed assessing the EWARS Mobile epidemiological surveillance tool toward humanitarian crises in Africa, more particularly in Chad. The purpose of this study was to evaluate the application of the EWARS Mobile epidemiological surveillance tool in Sudanese children's refugees in Eastern Chad at the provincial, district, and subdistrict levels, as well as EWARS Mobile indicator-based performance.

## 2 Methods

### 2.1 Study design

This was a retrospective and population-based surveillance study conducted in vulnerable children in Eastern Chad provinces of Ouaddaï, Sila, and Wadi Fira between 1 August and 1 December 2024.

### 2.2 Study population and settings

**Figure 1:**
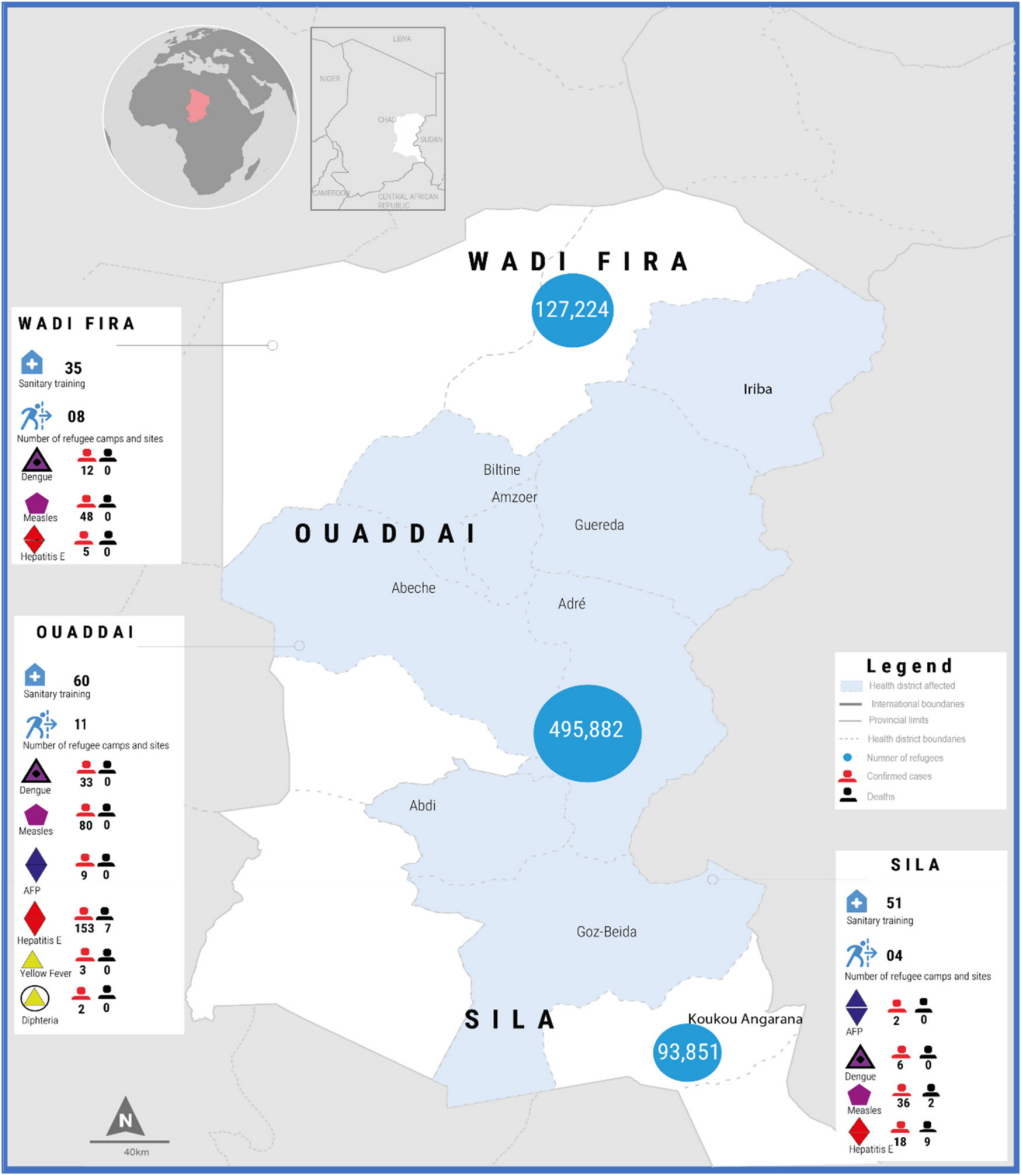
Map of the study area in Eastern Chad, including the provinces of Ouaddaï, Sila, and Wadi Fira.

The study included vulnerable children in different refugee camps in Eastern Chad, including the provinces of Ouaddaï, Sila, and Wadi Fira (Figure 1). The provinces of Ouaddaï, Sila, and Wadi Fira in Eastern Chad are home to hundreds of thousands of refugees from Sudan and are experiencing a health crisis. These refugees live in many formal and informal camps in nine health districts spread across Ennedi Est, Ouaddaï, Sila, and Wadi Fira provinces. However, in the camps, challenges in accessing essential health services remain due to multiple factors, including difficult physical access, limited medical supplies, and a lack of health and care workers. Flooding in affected regions has also added vulnerabilities by significantly destroying livelihoods, increasing the risk of waterborne diseases, and complicating response operations in the field (16). Less than 50% of the population in the eastern provinces has access to water, and only 33% of health facilities have a functional drinking water point, while the rate of open defecation is over 80% (1). Poor access to health services often leads to a worsening health situation. Malaria and ARI remain the most common diseases. Eastern Chad also faces the phenomenon of house and field fires during the dry season, which has a greater impact on household food security (1).

### 2.3 Description of EWARS Mobile and study procedures

WHO's EWARS Mobile is an application that improves disease epidemic detection in emergency situations, such as in conflict zones or after a natural disaster (5). WHO's EWARS Mobile is designed to improve disease outbreak detection in emergency settings, such as in countries in conflict or following a natural disaster. It is a simple and cost-effective way to rapidly set up a disease surveillance system (17). The main goal of EWARS Mobile during humanitarian crisis is to reduce excess morbidity and mortality from prioritized epidemic-prone diseases and other public health concerns.

EWARS Mobile consists of an online, desktop, and mobile application that can be quickly configured and deployed within 48 h of an emergency being declared. EWARS Mobile can be used in the following phases or processes: (1) Early warning employs EWARS mobile to collect data in preconfigured forms based on the monitoring strategy. Create automated information products, such as weekly epidemiological reports, depending on the data obtained. Provide regular feedback to reporting facilities, including report receipt, reminders for overdue reports, and immediate notifications when alerts are triggered, all via Short Message Service (SMS) (5). Health facilities are responsible for providing rapid notifications via designated phone and fax lines, as well as weekly reports. Weekly reporting is based on the normal epidemiological weeks. All instant notifications are received by the district surveillance office and recorded in alert logs for future monitoring and action. The district surveillance office receives weekly reports for aggregation in preparation for district-level analysis and subsequent transfer of district aggregate data to the national level (central) (18). (2) Alarm: After selecting alarm levels that are specific to your needs, EWARS Mobile will automatically send alerts when the threshold is surpassed. When an alarm is triggered, the EWARS Mobile can send an immediate SMS/email notification. If available, rapid response teams (RRTs) can intervene at the field level, as they are frequently the first to respond to alarms sent via EWARS Mobile (5). The datahub allows users to examine and update alerts for in-depth risk evaluations. In addition, the alert system can be coupled with laboratory surveillance (5). The findings of case investigations can be compiled and added to alerts. If laboratory samples have been obtained, laboratory users can update the results and send timely notifications to the field; (3) Response: Once an epidemic has been confirmed, EWARS Mobile can be programmed to collect case-based/line-list data, even while offline (5). Work can be done on the desktop app (with online) or datahub (without Internet) to create customized tables, charts, and maps. The analysis and/or raw data can be included into an automated information product, such as a daily epidemic notice. EWARS Mobile also allows you to redact identifying variables if you want to share anonymized raw data with partners (5).

**Figure 2:**
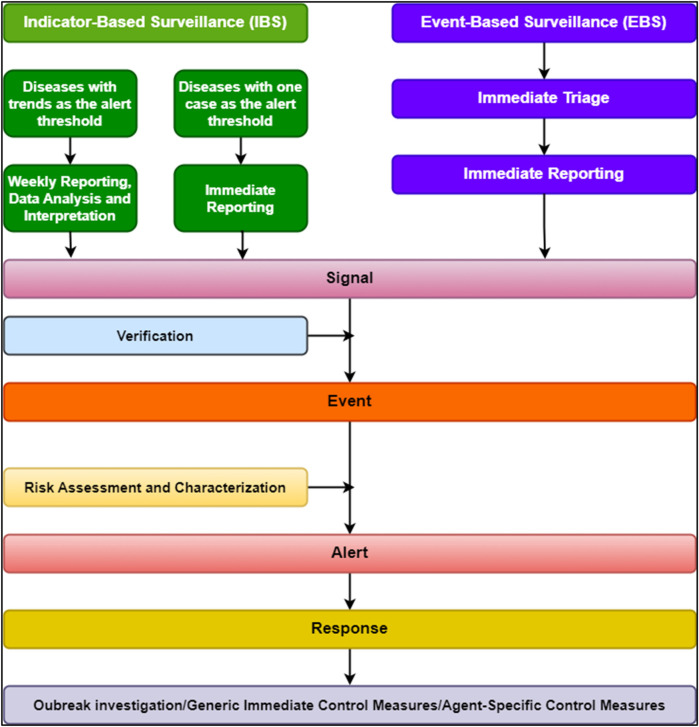
Flowchart showing the EWARS Mobile implementation and reporting cascade.

EWARS Mobile was initially deployed in November 2023 to address the Sudan refugee crisis in Eastern Chad. This phase concentrated on integrating service delivery indicators with early warning systems to manage health hazards among displaced people. The WHO, led by the Provincial Health Delegate, provided intensive training sessions. These lessons aimed to teach health workers how to utilize EWARS Mobile effectively, allowing them to issue situation reports and mobilize extra resources more swiftly. The deployment and execution of the EWARS Mobile for the Sudan refugee crisis is a multistep procedure that builds on previous experience while adjusting to current needs. The flowchart demonstrates the EWARS Mobile deployment and reporting cascade in Eastern Chad (Figure 2).

### 2.4 Data collection tool

In a typical early warning surveillance system, district levels regularly receive data from reporting sites. Data collection tool used EWARS Mobile indicators as described in the epidemiological tool (19). In a typical early warning surveillance system, district levels regularly receive data from reporting sites, as well as additional data on unusual health events from non-healthcare settings. Once received, the data from the different sources need to be compiled in one database to allow data analysis. Compilation can be done by merging data files from different sources if submitted electronically, or done manually by entering the data from the different reporting sources into one table or line list. Data collection, which was based on the country's routine surveillance system, has more recently been integrated by a more standardized protocol to ensure compliance and high-quality data reporting. Nonetheless, data quality was checked to ensure that at least 95% of the cells are complete with the correct information. Furthermore, to ensure that there were no discrepancies in the data, Stata 18 was used to check all the variables. In case of any discrepancies, revision of the original forms or discussion with data collectors was undertaken, followed by corrections and maintenance of the original file. Weekly information of potential key alarm indicators was collected from each study district/municipality.

We used the anonymized alert unit database, covering the health provinces of Ouaddaï, Sila, and Wadi Fira. Surveillance data were collected on relevant outbreak and alarm indicators covering data records for the period from 1 August 2024 to 1 December 2024. The following variables were extracted from the compiled Excel spreadsheet: alert ID, province, zone/camps, day of cases assessment, weeks of reporting, notification day, cases assessed, cases notified, total deaths, acute flaccid paralysis (AFP), acute jaundice syndrome (AJS), ARI, acute watery diarrhea in children (AWD) under 5 years, AWD among 5 years and older, measles, meningitis, diphtheria, dengue, neonatal tetanus (NT), bloody diarrhea, and unusual events.

### 2.5 EWARS Mobile performance indicators and definitions

Indicators for monitoring the performance of an EWARS Mobile surveillance system and calculation mechanism are described in the Supplementary Table S1 (18).

### 2.6 Statistical analysis

Statistical analysis was undertaken using Stata 18 MP software. To provide an overview of the pattern of cases and deaths related to AFP, AJS, ARI, acute AWD in less than and above 5 years old, measles, meningitis, diphtheria, NT, dengue, dysentery, and unusual events, descriptive statistics of both graphics and estimates presentations were undertaken. We also computed indicators for monitoring the performance of EWARS Mobile surveillance, including completeness of data recording, completeness of case reporting, completeness of alerts recording, timeliness of alert reporting, completeness of weekly reporting, timeliness of weekly reporting, timeliness of alert verification, and timeliness of outbreak investigation. An indicator with a performance below 80% was deemed to have performed inadequately (20). We also presented sensitivity, specificity, positive predictive values (PPV), and negative predictive values (NPV) and their 95% CI based on the number of alerts and outbreaks identified. In the same line, receiver operating characteristic (ROC) was also reported. Finally, epidemic curves were constructed to monitor the temporal trends of the aforementioned communicable diseases among vulnerable children.

### 2.7 Ethical consideration

Ethics clearance was obtained from the Chadian Ministry of Health, which was approved by local health authorities (Ethics approval number: 0988/MSP/SE/SG/2025). This study followed the Helsinki Declaration and local institutional rules on human research. Furthermore, the data were collected anonymously, and neither the tools nor the results contain the names of participants. The investigators obtained ethical approval and a waiver of consent from the ethics committee of Chadian Ministry of Health. Patients were not engaged in selecting the research question or the outcome measures, nor in the design and administration of the study. The confidentiality of the participants was maintained by assigning a unique code to each individual and securing the data in a locked cabinet and password-protected computer.

## 3 Results

### 3.1 Description of cases and deaths reported by province

Between 1 August and 1 December 2024, a total of 1,645 alarms were registered in vulnerable children in Quaddai, Sila, and Wadi Fira provinces. An estimated 91,528 cases were reported. There were 41,738 notified cases and 236 deaths, resulting in a 0.56% anticipated mortality rate. Quaddai reported 19,927 alerted cases and 108 fatalities; Sila reported 9,472 alerted cases and 113 deaths; and Wadi Fira province reported 12,339 alerted cases and 15 deaths.

Sila province had the greatest rate of alert AFP cases, with 55/65 (85%), followed by Wadi Fira province with 8/65 (12%). Sila province had the highest AJS alarm instances with 395/556 (71%). The total AJS case mortality rate was estimated to be 6.80%, with Sila having the highest death rate (7.30%). ARI cases were higher in Ouaddaï province (12,114/24,354, 49%) than other provinces. However, Sila recorded a high death ARI rate, estimated at 51/24,354 (0.21%) (Table 1). There were slight variances in AWD case reporting in under 5 years, with 4,186/10,420 in Quaddai, 3,022/10,420 in Sila, and 3,212/10,420 in Wadi Fira province. Ouaddaï province reported the highest AWD fatality rate in under 5 years (0.39%). AWD in 5 years or older was recorded in 1,951/3,942 (49%) Ouaddaï province, 299/3,942 (8%) Sila province, and 1,692/3,942 (43%) Wadi Fira (Table 1).

**Table 1:** Description alert case and deaths reported in vulnerable children in Quaddai, Sila, and Wadi Fira from 1 August to 1 December 2024.

Province	Issue		Death-to-case ratio
	Total alert deaths	Total alert cases	
AFP			
Ouaddaï	0	2	0.000
Sila	0	55	0.000
Wadi Fira	0	8	0.000
Total	0	65	0.000
Acute jaundice syndrome			
Ouaddaï	9	135	0.067
Sila	29	395	0.073
Wadi Fira	0	26	0.000
Total	38	556	0.068
Acute respiratory infection			
Ouaddaï	7	12,114	0.000
Sila	51	5,211	0.001
Wadi Fira	0	7,029	0.000
Total	58	24,354	0.002
Acute watery diarrhea under 5 years			
Ouaddaï	41	4,186	0.010
Sila	7	3,022	0.002
Wadi Fira	13	3,212	0.004
Total	56	10,420	0.005
Acute watery diarrhea 5 years and above			
Ouaddaï	8	1,951	0.004
Sila	0	299	0.000
Wadi Fira	0	1,692	0.000
Total	8	3,942	0.002
Measles			
Ouaddaï	8	10	0.800
Sila	0	8	0.000
Wadi Fira	0	33	0.000
Total	8	51	0.157
Meningitis			
Ouaddaï	8	8	1.000
Sila	0	0	0.000
Wadi Fira	0	0	0.000
Total	8	8	1.000
Diphtheria			
Ouaddaï	24	11	2.181
Sila	0	0	0.000
Wadi Fira	0	0	0.000
Total	24	11	2.181
Dengue			
Ouaddaï	0	0	0.000
Sila	0	0	0.000
Wadi Fira	0	0	0.000
Total	0	0	0.000
Neonatal tetanus			
Ouaddaï	0	1	0.00
Sila	13	3	4.33
Wadi Fira	0	1	0.000
Total	13	5	2.600
Dysentery			
Ouaddaï	2	759	0.003
Sila	11	450	0.024
Wadi Fira	2	238	0.008
Total	15	1,447	0.010
Unusual events			
Ouaddaï	1	745	0.001
Sila	2	21	0.095
Wadi Fira	0	100	0.000
Total	3	866	0.003

Ouaddaï province had 100% of measles deaths, meningitis, and diphtheria (Table 1). Interestingly, five occurrences of NT were reported, compared with 13 deaths. Furthermore, Quaddai, Sila, and Wadi Fira provinces recorded 52%, 31%, and 16% of dysentery cases, respectively. The dysentery for case fatality rate was significant in Sila province (0.76%) (Table 1).

### 3.2 EWARS Mobile performance indicators

Our results indicate that EWARS Mobile surveillance coverage was 100% in Ouaddaï, Sila, and Wadi Fira. Across the three provinces, case reporting, alert recording, and weekly reporting were all 100% complete. Table 2 also shows that Wadi Fira (66.55%) and Ouaddaï (66%) provinces lag behind Sila (87%) in terms of alert reporting timeliness. Ouaddaï, Sila, and Wadi Fira excelled at weekly reporting timeliness, scoring 87%, 99%, and 98%, respectively. Ouaddaï, Sila, and Wadi Fira had timely outbreak investigations with scores of 79%, 96%, and 89%, respectively (Table 2).

**Table 2:** EWARS Mobile performance indicators by province reported from 1 August to 1 December 2024.

Indicators	Ouaddaï	Sila	Wadi Fira	Total
Completeness of case reporting	100	100	100	100
Completeness of alerts recording	100	100	100	100
Timeliness of alert reporting	66	87	63	72
Completeness of weekly reporting	100	100	100	100
Timeliness of weekly reporting	87	99	98	95
Timeliness of outbreak investigation	79	96	89	88

In terms of the performance indicator by health district, our results showed that Amleyouna district reported poor performance timeliness of alert reporting (49%), timeliness of weekly reporting (72%), and timeliness of outbreak investigation (64%). Hadjer Hadid also reported poor performance for the same indicators, with 59%, 84%, and 75%. Iriba district (54%) and Guereda (79%) performed poorly in terms of timeliness of alert reporting (Table 3).

**Table 3:** Performance indicator by health district, site/zone, and camp reported in Eastern Chad from 1 August to 1 December 2024.

Location	Completeness of alerts reporting (%)	Timeliness of alert reporting (%)	Timeliness of weekly reporting (%)	Timeliness of outbreak investigation (%)
Health districts				
Adre	100	83	94	93
Amleyouna	100	49	72.	64
Chokoyane	100	89	100	100
Goz Beida	100	89	99	97
Guereda	100	79	98	93
Hadjer Hadid	100	59	84	77
Iriba	100	54	99	87
Koukou Angarana	100	81	98	95
Sites/zones				
Adre Urbain	100	61	77	73
Allacha	100	25	68	43
Am-nabak	100	81	99	93
Arkoum	100	69	96	82
Brejin	100	66	92	84
Camp Djabal	100	58	99	92
Camp Goz-Amir	100	90	100	100
Camp Kounoungou	100	95	100	100
Camp Mile	100	57	94	84
Camp Moura Kouchaguine	100	22	62	46
Chokoyane	100	89	100	100
Daguessa	100	71	97	89
Djoroko	100	85	96	95
Dogdore	100	100	100	100
Doroti	100	96	99	98
Farchana	100	74	97	93
Gaga Camp	100	84	85	88
Iridimi	100	36	99	84
Kerfi	100	87	100	96
Touloum	100	48	99	74
Trejin	100	26	57	32
Camps				
CS Daguessa	100	71	97	89
CS Dogdoré	100	100	100	100
CS Goz-Amir	100	80	100	100
Camp Allacha	100	16	69	43
Camp Am-Nabak	100	81	99	93
Camp Djabal	100	58	100	92
Camp Iridimi	100	36	99	84
Camp Kerfi	100	87	100	96
Camp Kounoungou	100	95	100	100
Camp Mile	100	57	94	84
Camp Touloum	100	48	99	75
Camp de Dougui	100	89	100	100
Camp de Gaga	100	84	85	88
Camps Metche	100	87	100	97
Doroti	100	95	99	97
Goz Achie	100	67	100	100
Kouchaguine-Moura	100	22	62	46
Site de transit Adré	100	48	100	77
Zabout	100	96	99	98

With regard to the performance indicator by site, our findings revealed that EWARS Mobile performed perfectly in terms of alert reporting completeness (Table 4). Adre Urbain performed poorly with 61%, 77%, and 73% in terms of the three timeliness indicators, as did Allacha district (25%, 68%, and 43%, respectively), Camp Moura Kouchaguine (22%, 62%, and 46%), and Trejin (26%, 57%, and 32%). Furthermore, Arkoum (69%), Brejin (66%), Camp Djabal (58%), Camp Mile (57%), Daguessa (71%), Farchana (74%), and Iridimi also scored poorly in terms of alert reporting timeliness. Touloum scored poorly on both alert reporting timeliness and outbreak investigation, with 49% and 75%, respectively (Table 3).

**Table 4:** Indicator-based surveillance by disease reported in Eastern Chad provinces from 1 August to 1 December 2024.

Outbreak	Completeness of alerts reporting (%)	Timeliness of alert reporting (%)	Timeliness of weekly reporting (%)	Timeliness of probable outbreak investigation (%)
AFP	100	64	91	34
Acute jaundice syndrome	100	71	88	84
Acute respiratory syndrome	100	68	92	86
Acute watery diarrhea less than 5 years	100	70	93	87
Acute watery diarrhea 5 years and above	100	64	93	84
Measles	100	70	93	76
Meningitis	100	100	100	100
Diphtheria	100	100	100	100
Neonatal tetanus	100	80	100	100
Dysentery	100	72	90	84

Our findings revealed that Camp Allacha scored poorly on the three timeliness indicators, with 16%, 69%, and 43% for alert reporting, weekly reporting, and outbreak investigation, respectively. The Kouchaguine-Moura camp showed similar results, with three timeliness indicators falling short of the 80% target (Table 3). CS Daguessa, Camp Djabal, Camp Iridimi, Camp Mile, and Goz Achie all had low alert reporting timeliness scores (less than 80%). Camp Touloum and Site de transit Adré both had low alert reporting and outbreak investigation timeliness scores (less than 80%) (Table 3).

### 3.3 EWARS Mobile performance indicators by alert disease

The completeness of alert reporting for all notifiable diseases in this study was 100% (Table 4). Conversely, timeliness of alert reporting varied between notifiable conditions, with 100% for both meningitis and diphtheria (Table 4). Timeliness of weekly reporting for acute jaundice syndrome was 88% and 100% for meningitis, diphtheria, and neonatal tetanus (Table 4). AFP, AJS, ARI, AWD under 5 years, AWD, measles, and dysentery cases were not reported timeliness of alert (less than 80%) (Table 4).

### 3.4 Description of cases and deaths alerted over time

**Figure 3:**
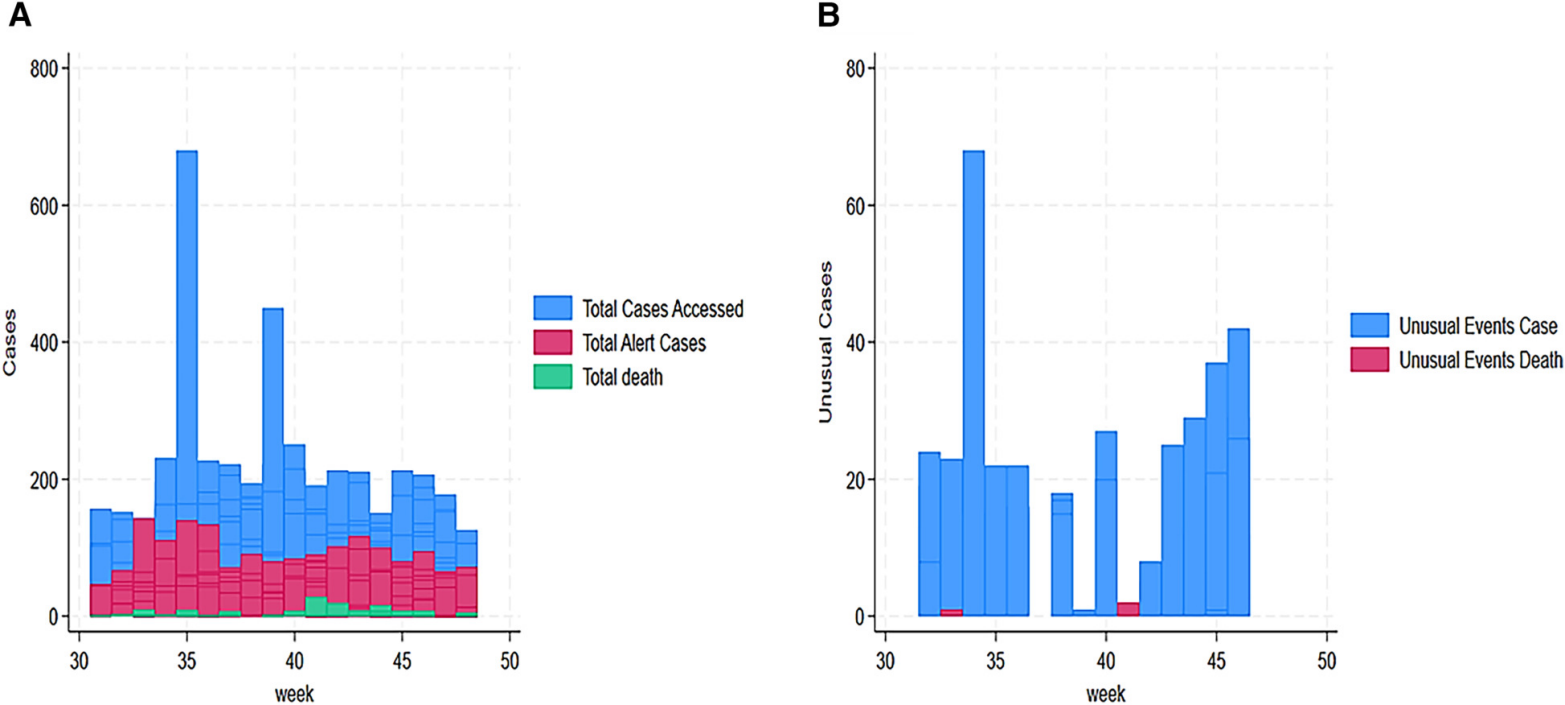
**(A)** Total cases accessed, alerted, and death in Sudanese children in Eastern Chad provinces. **(B)** Weekly evolution of unusual event cases and deaths in Sudanese children in Eastern Chad provinces.

Figure 2 shows the trend of cases assessed, reported, alerted, and deaths from week 31 to 48. The trend showed that high assessed cases were reported in week 35 (680 alerts), week 39 (450 alerts), and week 40 (251 alerts). The trend also showed that 143 alerted cases were reported in week 33, 140 cases in week 35, 134 cases in week 36, and 117 in week 43. In addition, weeks 42 and 43 reported the highest death cases with 19 and 8, respectively (Figure 3A). Unusual event cases and deaths reached a peak of 68 cases in week 34; however, deaths associated with unusual event cases were estimated at 3 over time (Figure 3B).

**Figure 4:**
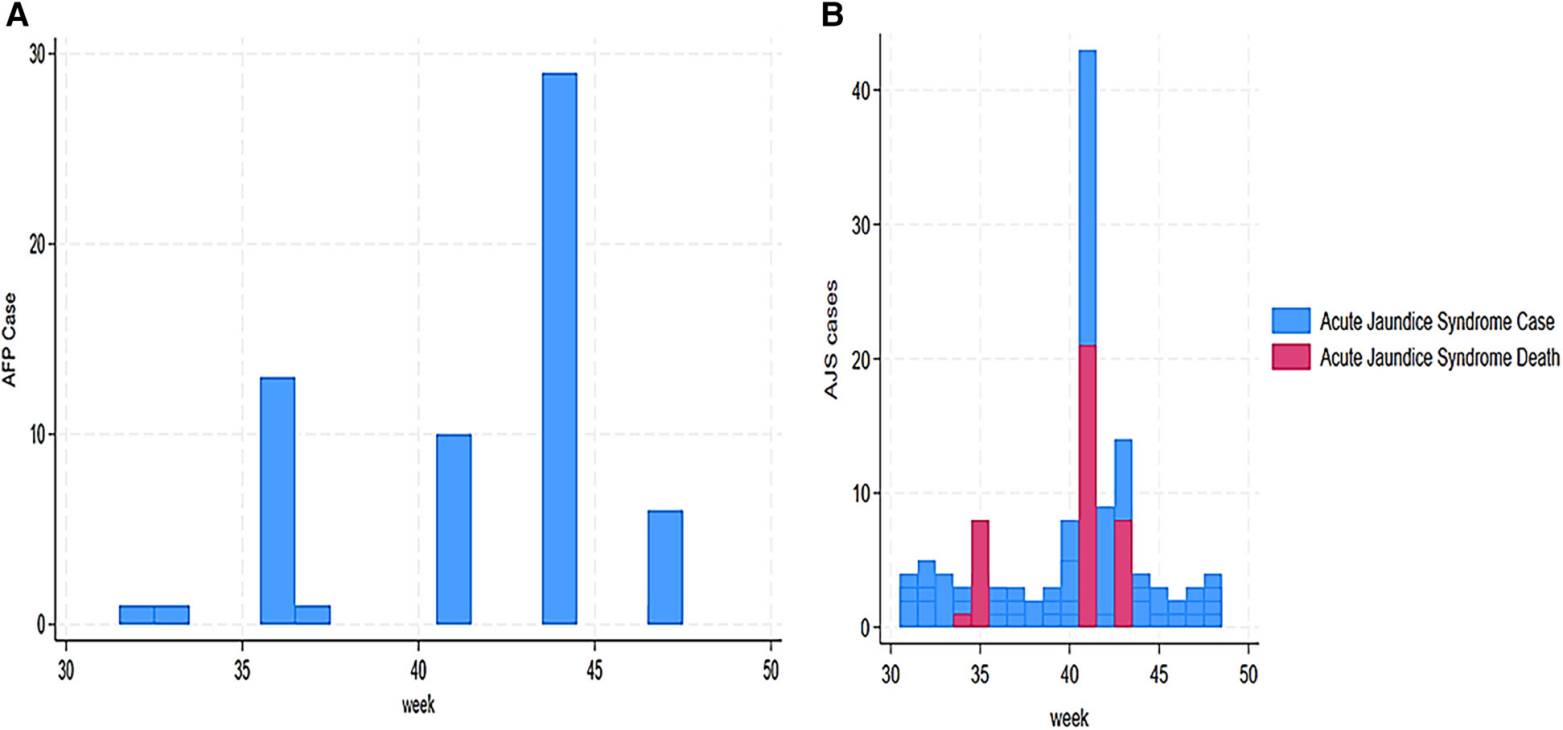
**(A)** Weekly evolution AFP cases in Sudanese children in Eastern Chad provinces. **(B)** Weekly evolution AJS cases and deaths in Sudanese children in Eastern Chad provinces.

**Figure 5:**
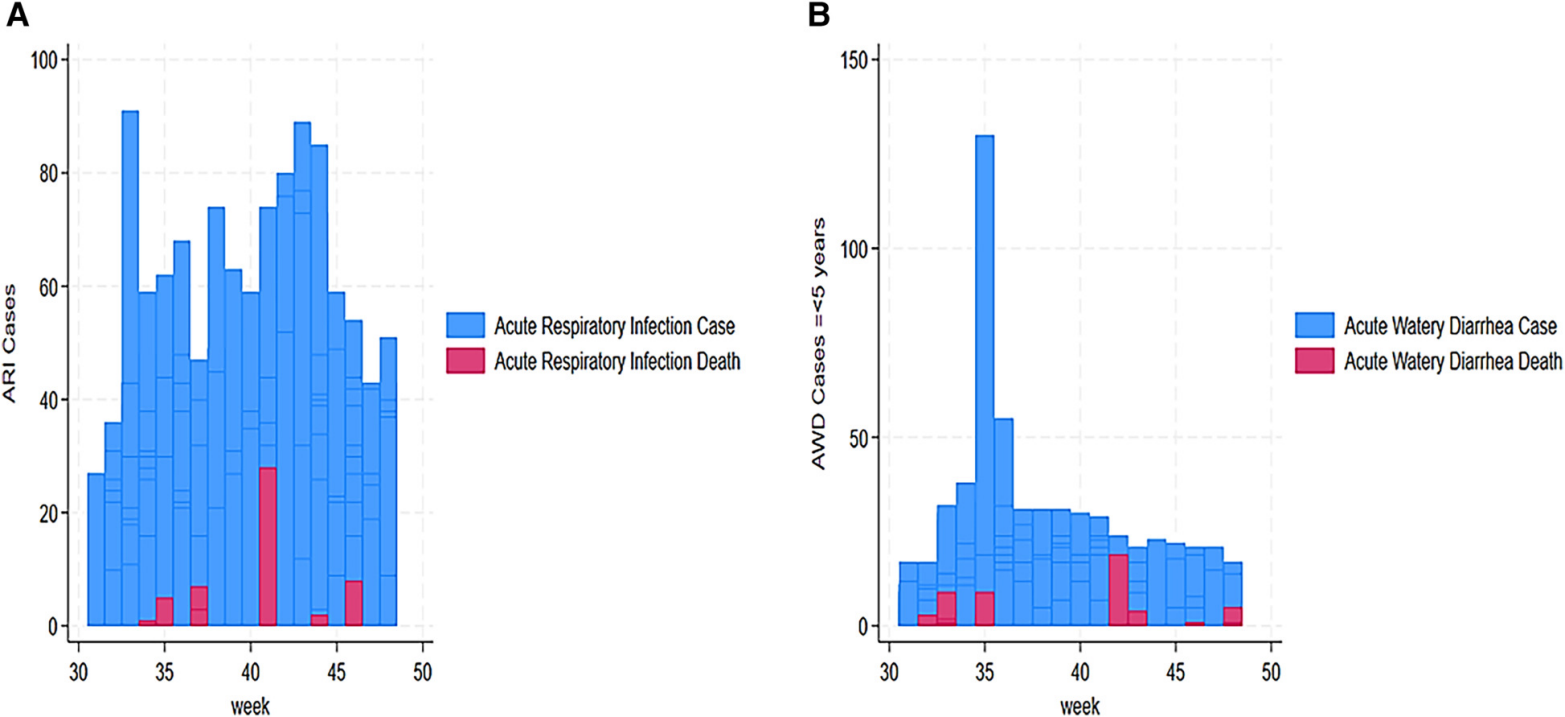
**(A)** Weekly evolution of ARI cases and deaths in Sudanese children in Eastern Chad provinces. **(B)** Weekly evolution of AWD in less than 5 years cases and deaths in Sudanese children in Eastern Chad provinces.

**Figure 6:**
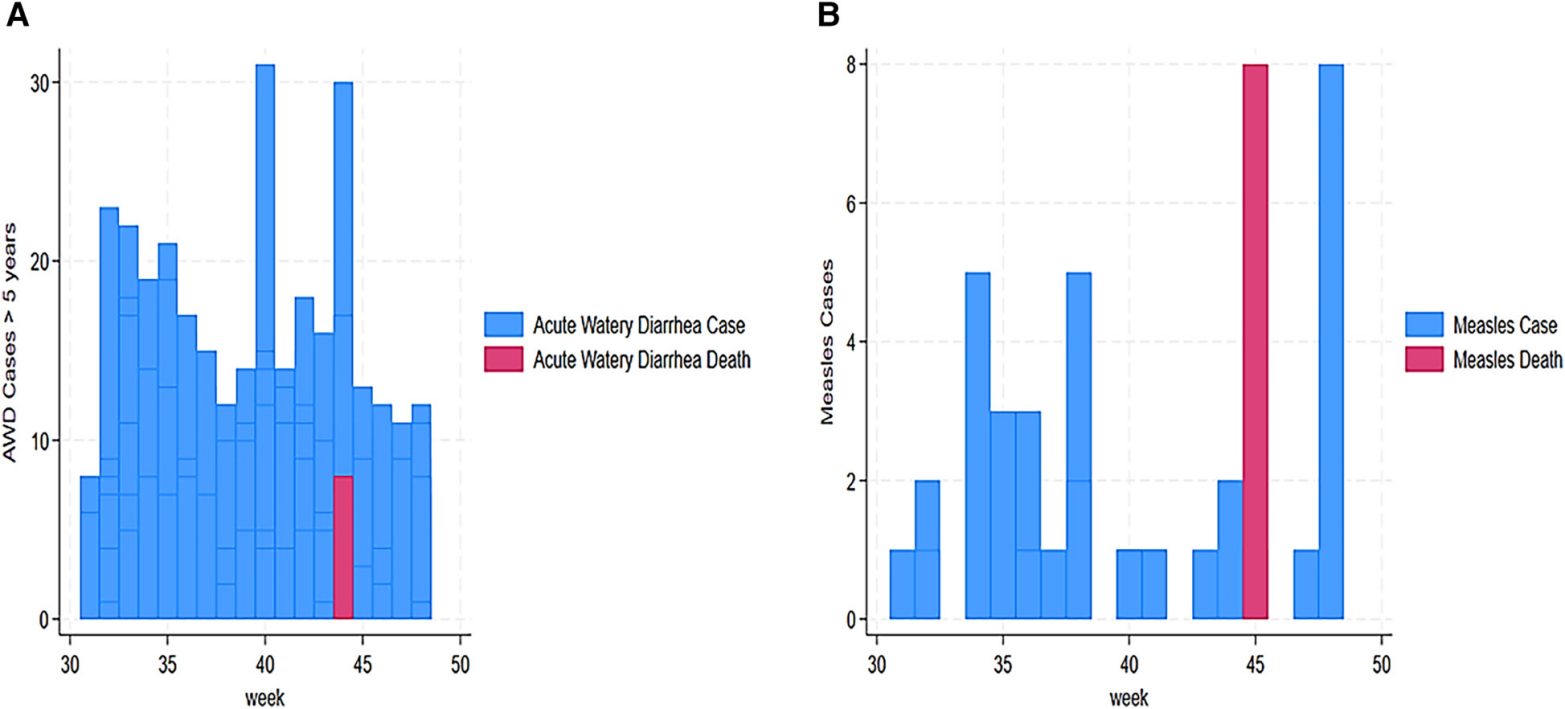
**(A)** Weekly evolution of cases and deaths of AWD in 5 years and above in Sudanese children in Eastern Chad provinces. **(B)** Weekly evolution of measles cases and deaths in Sudanese children in Eastern Chad provinces.

**Figure 7:**
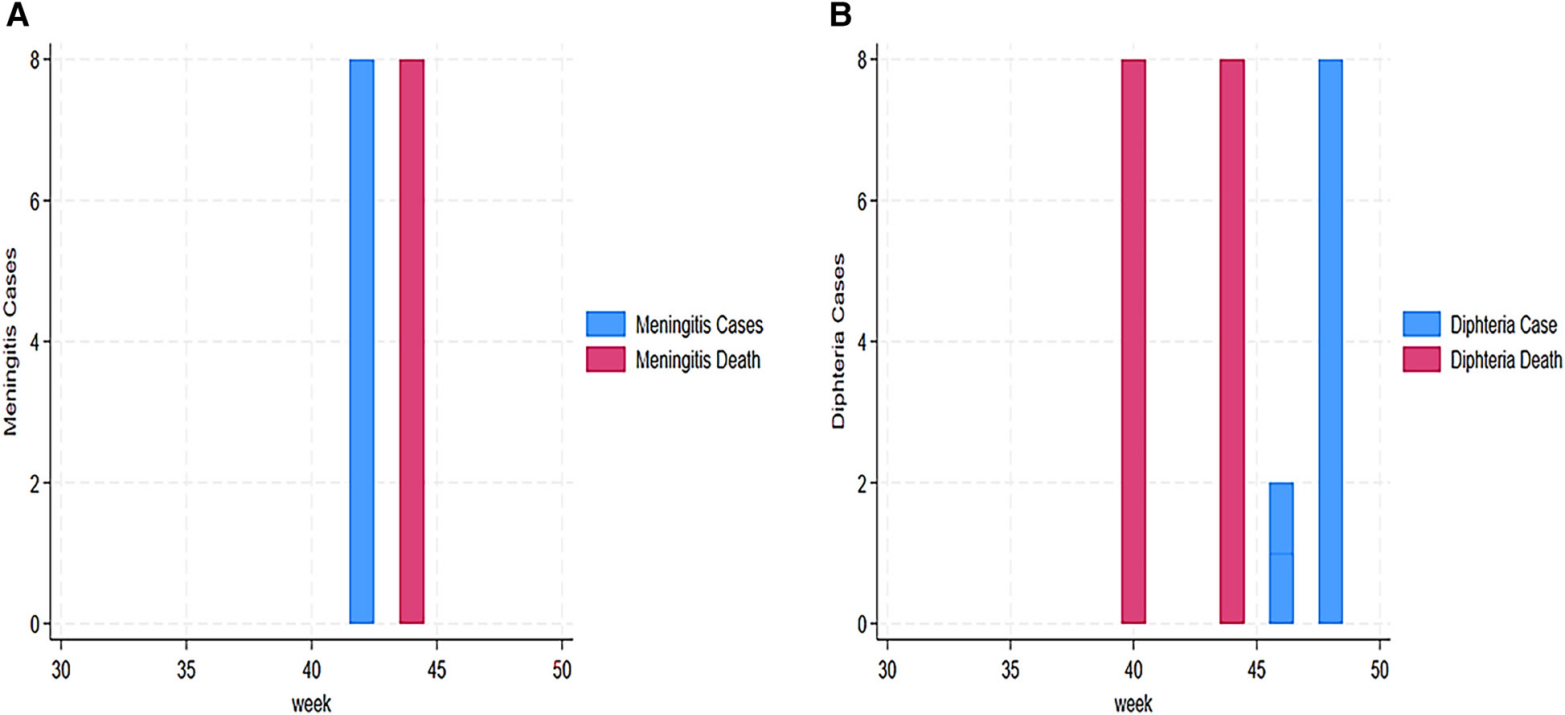
**(A)** Weekly evolution of meningitis cases and deaths in Sudanese children in Eastern Chad provinces. **(B)**) Weekly evolution of diphtheria cases and deaths in Sudanese children in Eastern Chad provinces.

**Figure 8:**
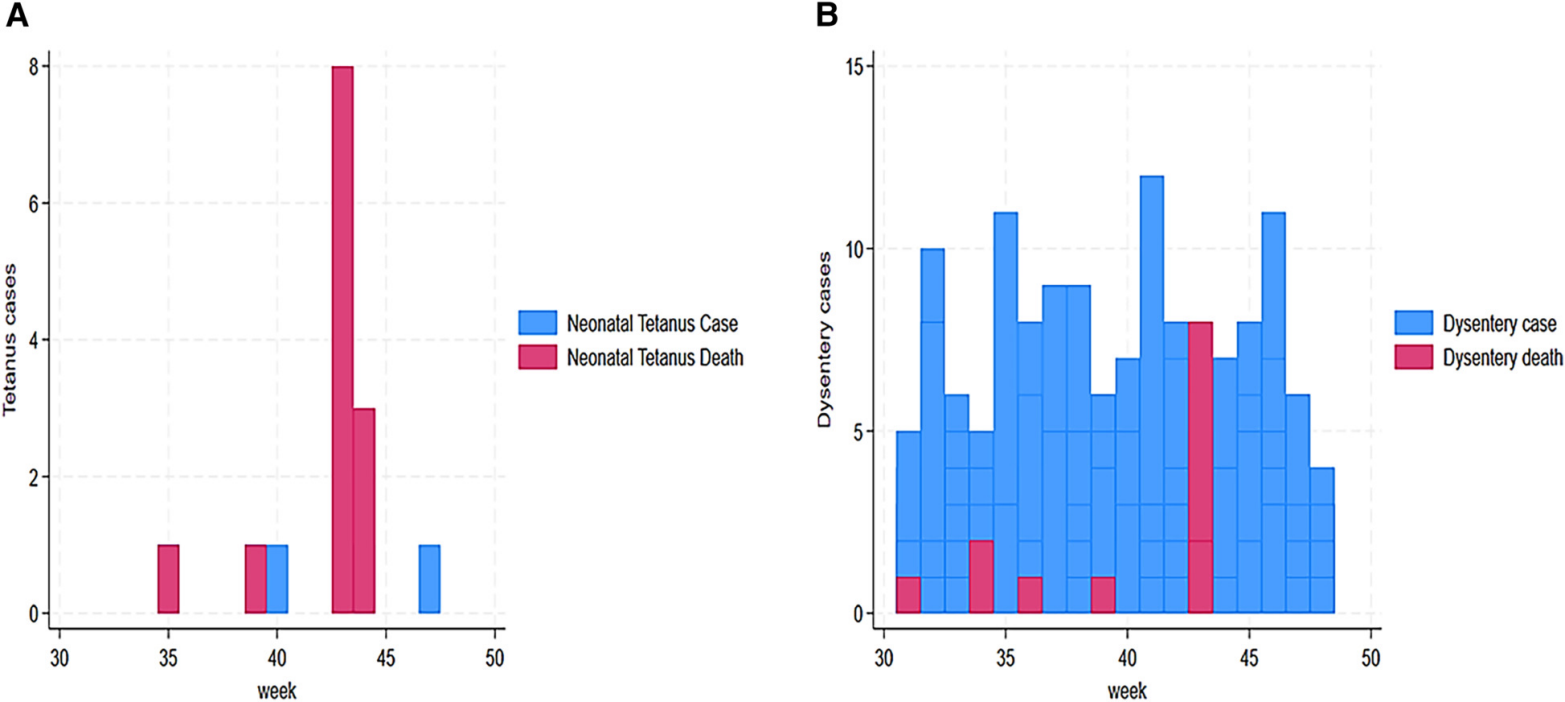
**(A)** Weekly evolution of NT cases and deaths in Sudanese children in Eastern Chad provinces. **(B)** Weekly evolution of dysentery cases and deaths in Sudanese children in Eastern Chad provinces.

Our findings also revealed a trend of AFP alert cases from 31 to 48 weeks. This trend indicated a high number of AFP cases in week 44 (29 cases), followed by weeks 36 and 41 with 13 and 10 cases, respectively (Figure 4A). Our findings demonstrated that AJS cases and mortality were the highest in weeks 41 and 43. We assessed cases vs. deaths at 43 vs. 21 in week 41 and 14 vs. 8 in week 43 (Figure 4B). Furthermore, eight deaths from AJS were reported in week 35 (Figure 4B). The weekly development of ARI cases revealed a large number of them, ranging from 27 (week 31) to 91 (week 33) (Figure 5A). Similarly, ARI caused 7, 28, and 8 deaths in weeks 37, 41, and 46, respectively (Figure 5A). In terms of the weekly evolution of AWD in under 5 years, we observed an increase in cases in weeks 33, 34, 35, and 36, with 32, 38, 130, and 55, respectively. AWD in under 5 years resulted in 9, 9, and 19 deaths in weeks 33, 35, and 42, respectively (Figure 5B). The trend of AWD in children aged 5 and up was high in weeks 40 and 44, with 31 and 30 occurrences, respectively (Figure 6A). Similarly, week 44 reported eight deaths in children aged 5 and up due to AWD (Figure 6A). The trend of measles was characterized by two peaks, mainly eight deaths recorded in week 45 and eight cases recorded in week 48 (Figure 6B). Eight cases of meningitis were reported in week 42 and all of them died in week 44 (Figure 7A). The trend of diphtheria showed 16 deaths in 40 and 44 weeks, and cases were still recorded in 46 and 48 weeks (Figure 7B). Figure 8A shows a high number of NT deaths (11) in weeks 43–44. Figure 8B shows the trend of dysentery cases and deaths, characterized by multiple peaks between 4 and 12 cases and reaching eight deaths in week 43. Epidemic curves showed many potential outbreak types with a point source (AJS and AWD under 5 years), a common source (AWD in 5 years or more), a propagating source (ARI and dysentery), and an intermittent source (AFP, measles, meningitis, diphtheria, NT, and unusual events) (Figures 3A–8B).

### 3.5 EWARS Mobile performance

**Figure 9:**
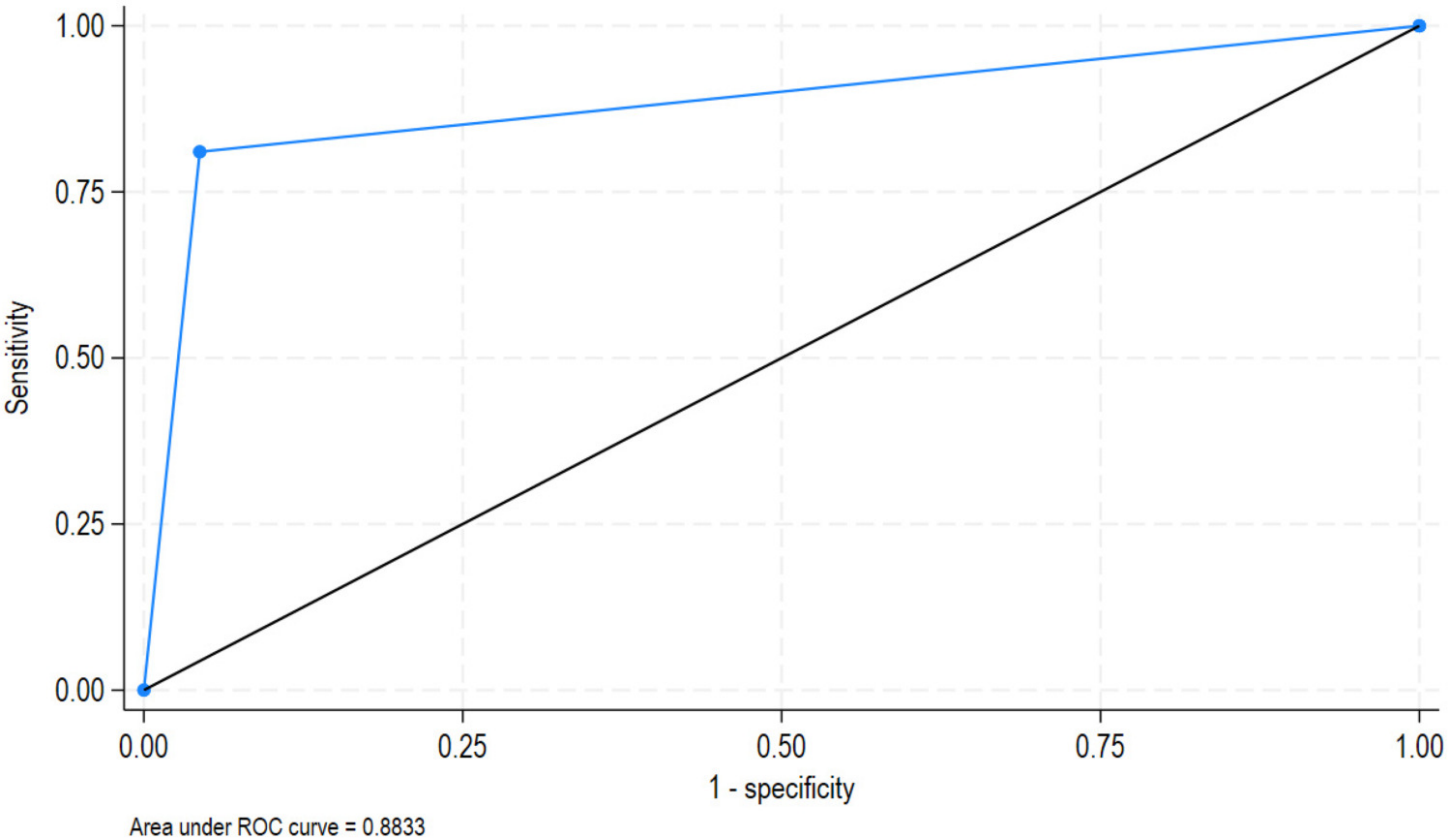
EWARS Mobile performance showing the AUC.

Based on the number of the alert and outbreaks reported, we computed sensitivity (95% CI) of 81% (78.9%–83%), specificity (95% CI) of 96% (92%–98%), PPV (95% CI) of 72.00% (68%–75%), and NPV (95% CI) of 50.2% (45.8%–54.8%). Furthermore, the area under the curve (AUC) was estimated at 88% (87%–90%) (Figure 9).

## 4 Discussion

This study sought to assess the deployment of the EWARS Mobile epidemiological monitoring tool in Sudanese children refugees' camps in Eastern Chad, encompassing province, district, and subdistrict levels, along with performance based on EWARS Mobile indicators from 1 August to 1 December 2024. According to our findings, EWARS Mobile was able to identify multiple concomitant alert probable outbreaks, including AFP, AJS, ARI, and AWD in under and above 5 years’ old, and measles, meningitis, diphtheria, NT, dengue, dysentery, and unusual events in children. Epidemic curves indicated multiple probable outbreak types, characterized by a point source (AJS and AWD in under 5 years), common source (AWD in 5 years and above), propagated source (ARI and dysentery), and intermittent source (AFP, measles, meningitis, diphtheria, NT, and unusual events). This was in line with a statement that stipulates that disease outbreaks do not occur in isolation from one another, and their reach is often compounded by pre-existing vulnerabilities, mainly in the case of humanitarian crises in Eastern Chad (21). Chad is the country most affected by Sudan's turmoil, hosting 45.7% of Sudanese refugees and thousands of new arrivals each week in Eastern Chad. According to our data, 1,645 alarms were reported among vulnerable children in Quaddai, Sila, and Wadi Fira provinces between 1 August and 1 December 2024. An estimated 91,528 cases were documented, with 41,738 notified patients and 236 deaths, for a forecasted mortality rate of 0.56%. The mortality rates might be underestimated due to incomplete data or lack of access to healthcare. This mortality rate may be lower than the humanitarian catastrophe in Sudan, where a report found a crude mortality rate of 2.5 per 10,000 people per day in children (22). Our findings showed that high alerts on acute jaundice syndrome, acute respiratory infection, acute watery diarrhea in under and above 5 years old, and measles are shown in the trend figures. Knowing that ARI and acute watery diarrhea are the leading causes of mortality in children in sub-Saharan Africa (23), identifying these causes by using EWARS Mobile in humanitarian crises in Eastern Chad was critical to minimizing children mortality. This approach was in line with the statement stipulating that understanding the needs of crisis-affected persons and orchestrating rapid response play decisive factors in the effectiveness of humanitarian aid. Innovative technology and products can enhance the provision and quality of humanitarian assistance to contend with these growing challenges (24–26).

Our findings revealed that EWARS Mobile performed well in terms of completeness of case reporting, alert recording, and weekly reporting. However, the timing of alarm reporting, weekly reporting, and epidemic investigation performance varied widely across provinces, districts, zones, and camps. However, the high completeness may result from WHO's involvement rather than the tool's efficacy, while the timeliness concerns could arise from logistical challenges in refugee camps rather than the tool itself. In Ouaddaï and Wadi Fira provinces, Amleyouna, Hadjer Hadid, Iriba, and Guereda districts, as well as Adre Urbain, Allacha district, Camp Moura Kouchaguine, Trejin, Arkoum, Brejin, Camp Djabal, Camp Mile, Daguessa, Farchana, and Iridimi zones, at least one timeline was below 80%. Sites and districts with all three EWARS Mobile timeliness indicators below 80% call for a public action to identify gaps and obstacles in using EWARS Mobile. These locations and camps included Adre Urbain, Allacha district, Camp Moura Kouchaguine and Trejin (districts), and Camp Allacha Kouchaguine-Moura (camps). The inconsistency in performance across regions warrants elucidation or hypotheses. This may be attributed to deficient health systems in these particular areas, as well as inadequate logistics and resources. Incorporating qualitative data into the EWARS Mobile regarding usability, training challenges, and user satisfaction could yield actionable insights for system enhancement. In addition, EWARS Mobile is shown as a successful example of innovative technology in humanitarian response with a positive impact in Eastern Chad. Even though these large-scale displacements have radically transformed the public health landscape in Eastern provinces of Chad, increasing the risk of disease outbreaks and complicating the delivery of essential health services (4), EWARS Mobile has shown its performance on communicable disease surveillance in children in severe humanitarian crises. It provides a functional and simple digital surveillance system and is rapidly implemented in an emergency setting for timely detection of and response to new outbreaks at all subnational levels, as shown in this study. It has succeeded in disease monitoring, detecting outbreaks, and driving public health action (24, 27, 28). A study reported that EWARS Mobile users were satisfied with the performance of EWARS Mobile and judged it useful for timely monitoring of disease trends and outbreak detection (29). The system was simple, stable, and flexible and could be rapidly deployed during a health emergency. The automated collation, analysis, and dissemination of data reduced the burden on surveillance teams, saved human resources, minimized human error, and ensured that teams could focus on public health responses (29). EWARS Mobile provided timely information on epidemic potential and preventable diseases in children. EWARS Mobile helped in targeting new vaccination campaigns and investigating suspected outbreaks (12, 24, 30). The sensitivity rate of correctly predicted outbreaks was 81% (79%–83%) and a PPV (95%CI) of 72.0% (68%–75%). Our findings were not so different from EWARS Mobile used to assess dengue outbreaks in different countries; the sensitivity of correctly predicting an outbreak ranged between 83% and 99% in Brazil, 50% and 99% in Malaysia, 79% and 100% in Mexico (30), and 97% in Colombia (13). In the same vein, PPVs ranged between 40% and 88% in Brazil, 71% and 80% in Malaysia, 50% and 83% in Mexico (30), and 75% in Colombia (13). The ROC curve provides a valuable tool for evaluating the performance of diagnostic tests used in EWARS mobile surveillance. By plotting the true-positive rate against the false-positive rate, it allows researchers to assess how well the system can identify specific events. Based on the AUC of 88%, EWARS Mobile should be considered a good or excellent tool to implement throughout the pre-epidemic and outbreak periods in vulnerable children in this severe humanitarian crisis.

This is the first study undertaken in Chad to evaluate the EWARS Mobile epidemiological monitoring tool among Sudanese children's refugees. This study showed that EWARS Mobile may be used in all subnational areas to predict outbreaks in humanitarian crises. The use of important surveillance indicators, including completeness and timeliness at all levels, is also the strength of this study. Furthermore, the study presented all the alerted conditions both in space and time. The completeness and timeliness of the reporting system in each indicator were classified as a score of ≥80%: “sufficient,” in line with the WHO JEE Tool, which recommends completeness and timeliness of reporting at least 80% of all reporting units (31, 32). In addition, this study includes multiple limitations. Because of its retrospective character, this study had missing data. Furthermore, we cannot exclude selection and misclassification biases. The data did not provide adequate sociodemographic characteristics of the studied population. The lack of sociodemographic data makes it difficult to appropriately determine disease vulnerability. Furthermore, qualitative data and those relative to the mean alarm indicator were missing. Knowing that poor disease surveillance has been attributed to a lack of experienced healthcare workers (HCWs), heavy workloads, and staff shortages, qualitative data would bring more clarity on timeliness reporting across refugee camp sites. As the momentum to scale up the worldwide response to communicable diseases grows, public health practitioners must regularly examine their effectiveness in detecting and responding to infectious diseases (8). Early warning systems have significant advantages in timeliness of reporting, flexibility to incorporate new syndromes of concern, and low administrative and laboratory burden. However, there are equally well-recognized limitations, including low specificity and positive predictive value and high false-alarm rates, which contribute to difficulties identifying true departures from statistical norms for outbreak detection purposes (28, 33). However, the success in detecting outbreaks could be attributed to increased vigilance rather than the tool's sensitivity. Finally, several meteorological and environmental indicators were used as predictors, of which temperature and rainfall were the most frequently used indicators, as they could be associated with outbreaks (30). A longer monitoring period for the second phase can provide more trustworthy data on seasonal fluctuations and the stability of the surveillance system. In addition, challenges were also observed, which were mainly insufficient resources, such as a lack of a network to use general packet radio service (GPRS) coordinates, a lack of confirmed diagnoses, and a scarcity of vaccines and medicines.

## 5 Conclusion

The EWARS Mobile approach is a practical solution for Eastern Chad province to implement throughout the preoutbreak and outbreak phases in children affected by the extreme humanitarian crisis. EWARS Mobile system was able to send alerted probable outbreaks including AFP, AJS, ARI, and AWD in less and above 5 years old, measles, meningitis, diphtheria, NT, dengue, dysentery, and unusual events in children at all subnational levels. Even while the completeness performed well, the timeliness indications differed among settings. These sites and camps, including Adre Urbain, Allacha district, Camp Moura Kouchaguine and Trejin (districts), and Camp Allacha Kouchaguine-Moura (camps), require inspections and evaluations in the use of EWARS Mobile tool in humanitarian crisis because they all performed below the 80% target in all timeliness indicators. In the same vein, the sensitivity, PPV, and AUC demonstrated good performance. Moreover, efforts should be made to improve timeliness indicators at all subnational levels and to include alarm indications for outbreak identification.

## Data Availability

The raw data supporting the conclusions of this article will be made available by the authors without undue reservation.
