# Supplementary material for: Evaluation of the implementation of the EWARS Mobile epidemiological surveillance tool in Sudanese refugee camps in Eastern Chad: a retrospective and population-based surveillance study
Source: Front Epidemiol. 2025 Jul 23;5:1604446. doi: 10.3389/fepid.2025.1604446 (PMC12325338; doi:10.3389/fepid.2025.1604446)
Supplement: Supplementary file 1 [file Table1.docx]

**Supplemental Table 1: Core indicators on EWARS Mobile performance**

| N | Indicators | Calculations and definitions | Targets |
| --- | --- | --- | --- |
| 1 | Completeness of data recording | Number of filled surveillance forms that have all variables completed/all filled forms × 100 | ≥ 80% |
| 2 | Completeness of case reporting | Number of cases in filled surveillance forms for each reportable disease/ number of cases of the disease registered in the outpatient records × 100 | ≥ 80% |
| 3 | Completeness of alerts recording | Number of alerts recorded in the logbook/number of reported alerts in health facility records × 100 | ≥ 80% |
| 4 | Timeliness of alert reporting | Number of alerts reported within 24 hours of detection/total number of alerts × 100 | ≥ 80% |
| 5 | Completeness of weekly reporting | Number of reporting sources that reported data last week/all reporting sources × 100 | ≥ 80% |
| 6 | Timeliness of weekly reporting | Number of reporting sources that reported on time last week/all reporting sources × 100 | ≥ 80% |
| 7 | Timeliness of probable outbreak investigation | Number of probable outbreaks investigated within 72 hours/total number of outbreaks × 100 | ≥ 80% |
| 8 | Sensitivity | The ability of a surveillance or reporting system to detect all true health events. | ≥ 80%: Acceptable |
| 9 | Specificity | The ability of a surveillance or reporting system to exclude events that are not, in fact, true health events. | ≥ 80%: Acceptable |
| 10 | Positive Predictive Value (PPV) | This indicates how often a positive system alert result correctly identifies true health events. | ≥ 70%: Good performance |
| 11 | Under the Curve (AUC) | A fundamental tool for evaluating the performance of diagnostic tests. | ≥ 80%: Good performance |
